# Ciprofloxacin Controlled-Solid Lipid Nanoparticles: Characterization, In Vitro Release, and Antibacterial Activity Assessment

**DOI:** 10.1155/2017/2120734

**Published:** 2017-01-17

**Authors:** Gamal A. Shazly

**Affiliations:** ^1^Department of Pharmaceutics, College of Pharmacy, King Saud University, P.O. Box 2457, Riyadh 11451, Saudi Arabia; ^2^Department of Industrial Pharmacy, Faculty of Pharmacy, Assiut University, Assiut 71526, Egypt

## Abstract

The objective of this research was to formulate ciprofloxacin (CIP) in solid lipid nanoparticles (SLNs) in an attempt to develop a controlled drug delivery system. An ultrasonic melt-emulsification method was used for preparing CIP-loaded SLNs. Key findings included that SLNs were successfully produced with average particle sizes ranging from 165 to 320 nm and polydispersity index in the range of 0.18–0.33. High entrapment efficiency values were reported in all formulations. The atomic force scanning microscopic images showed spherical shape with the size range closer to those found by the particle size analyzer. CIP release exhibited controlled-release behavior with various lipids. Ciprofloxacin solid lipid nanoparticles formula containing stearic acid (CIPSTE) displayed the strongest burst effect and the most rapid release rate. The release data revealed a better fit to the Higuchi diffusion model. After storing the CIPSTE formula at room temperature for 120 days, no significant difference in particle size and zeta potential was found. CIP-loaded SLNs exhibited superior antibacterial activity. Incorporation of CIP into SLNs leads to controlled release and a superior antibacterial effect of CIP.

## 1. Introduction

Ciprofloxacin (CIP) is a broad spectrum fluoroquinolone antibiotic. CIP is used systematically in numerous microbial contaminations such as dermal, pulmonary, and urinary tract infections and topically in conjunctivitis [1] and anterior ocular infections [2]. CIP can be taken orally or intravenously. CIP was the most used antibacterial agent worldwide and the fifth regularly used generic antibacterial in the USA during the last decade in the 20th century [3]. CIP is active in the elimination of many gram-negative and some gram-positive bacteria, such as strains of bacterial pathogens causing abdominal, urinary tract, gastrointestinal, and respiratory infections. CIP is frequently used for prophylaxis and treatment of osteomyelitis caused by* P. aeruginosa* [4]. The low drug solubility problem can be avoided by formulating the drug in colloidal drug carriers, such as liposomes, micelles, polymeric nanoparticles, or nanosuspensions [5]. Conversely, these systems are accompanied by several problems like aggregation, drug leakage, limited physical stability, or the presence of organic solvent residues in the final product in storage [6]. To minimize these problems, a lipid-based drug delivery system can be used [7].

Solid lipid nanoparticles (SLNs) are novel drug delivery carriers having the advantages of both polymeric nanoparticles and liposomes [8, 9]. SLNs are composed of nanosized biodegradable and biocompatible lipid base carriers, which are predominantly solid at body and room temperature [5, 10]. Several studies have revealed that SLNs have high drug loading for both hydrophilic and lipophilic drugs [11], large-scale production [12], and long-term shelf stability [5]. Additionally, SLNs comprised of small particles from 120 to 200 mm are infrequently subject to blood clearance by the reticuloendothelial system in the liver and spleen [13]. SLNs are flexible nanocarriers and are used for drug delivery in nearly all routes of administration, including ocular [14], parenteral [15], peroral [16], and dermal [17]. SLNs can be formulated to sustain the drug release profile and therefore decrease the necessity for the repeated administration and enhance the therapeutic value of the treatment [5].

Previous studies formulate CIP SLNs for enhancing its therapeutic effect and improving its bioavailability [18, 19].

The aim of this study was to load CIP in SLNs formulations with high loading capacity, sustain the drug release, and study the in vitro antibacterial effect of the loaded CIP using different lipids.

## 2. Materials and Methods

### 2.1. Materials

The lipids Softisan® 154 (SOF), Dynasan® 118 (DYN), and Imwitor® 900 K (IMW) were bought from Germany (Sasol Germany GmbH, Witten, Germany). Tween 80, stearic acid (STE), and sodium deoxycholate were purchased from Sigma-Aldrich Chemical Company (St. Louis, MO, USA). All other materials were used as received and were of analytical grade.

### 2.2. Preparation of CIP SLNs

CIP SLNs were prepared using the ultrasonic melt-emulsification method as reported before (18), with some modification. Briefly, the lipid phase was prepared by melting specific weights of lipids (Softisan® 154 (SOF), Dynasan® 118 (DYN), and Imwitor® 900 K (IMW), Sasol Germany GmbH, Witten, Germany) and stearic acid (STE), Sigma-Aldrich Chemical Company, USA, above their melting points by about 10°C, and then, known weights of CIP were dissolved in the molten lipids. The aqueous phase was prepared by heating a mixture of 1% tween 80 (Sigma-Aldrich Chemical Company, USA) and 0.5% sodium deoxycholate (Sigma-Aldrich Chemical Company, USA) in distilled water to the same temperature as the lipid phase. Next, the aqueous phase was sonicated using a probe sonicator (Bandelin Sonopuls HD 220, Bandelin Electronics, Berlin, Germany) together with the lipid phase at 35% voltage efficiency for 4 minutes. The produced emulsion was stirred in chilled distilled water containing 1% glucose using a magnetic stirrer for 5 minutes (Table 1). The produced emulsion was stored at 4°C for further investigations.

### 2.3. HPLC Assay of CIP

A validated HPLC method was used for analyzing CIP release [20]. The HPLC system (Waters™ 600 controller, USA) is composed of a pump (Waters 1252 a Binary pump, USA) and UV detector (Waters 2487 a Dual *λ* Absorbance detector, USA) and equipped with an automated sampling system (Waters 717 PlusAutosampler, USA). The system was monitored by Empower (Water) software. CIP was examined using a mobile phase composed of buffer 0.025 M orthophosphoric acid (pH 2.1): methanol: acetonitrile at a ratio of 50 : 15 : 35. The mobile phase flowed over a reversed-phase C18 column (Bondapak™, 10 *μ*m particle sizes, 4.6 × 150 mm, Waters) at a rate of 1.5 mL/min. 20 *μ*L of CIP emulsion was injected and the amount of CIP released was detected at a wavelength of 275 NM. The assay was performed at room temperature.

### 2.4. Evaluation of SLNs

#### 2.4.1. Particle Size and Polydispersity

The mean particle size, particle size distribution, and polydispersity index of the prepared SLNs formulations were measured using Photon Correlation Spectroscopy (Brookhaven Instruments Corporation, Holtsville, NY, USA). The SLNs were diluted with distilled water (1 : 100) and the measurements were done at 25°C at an angle of detection of 90°.

#### 2.4.2. Zeta Potential

The particle size analyzer (A 90 Plus particle size analyzer, Brookhaven Instruments Corporation, Holtsville, NY, USA) was utilized for measuring the zeta potential of the prepared CIP SLNs. The samples were diluted with water in a ratio of 1 : 100 before measurement.

#### 2.4.3. Nanoparticle Yield, Drug Loading, and Entrapment Efficiency

The samples were subjected to centrifugation for 30 minutes at 4°C and 50,000 rpm using an ultracentrifuge (Optima MAX-E ultracentrifuge, Beckman Coulter, Inc., Nyon, Switzerland). The unencapsulated amount of CIP in the supernatant was analyzed using HPLC. Percent drug entrapment efficiency, drug loading, and nanoparticle yield of the prepared CIP SLNs were calculated using (1)–(3), respectively:

(1) Entrapment efficiency(%)=Weight of drug in nanoparticlesWeight of drug fed initially×100.

(2) Drug loading (%)=Weight of drug in nanoparticlesWeight of nanoparticles×100.

(3) Nanoparticle yield (%)=Weight of nanoparticlesWeight of lipid and drug fed initially×100.

#### 2.4.4. Scanning Electron Microscopy (SEM) Studies

The surface morphology of the prepared SLNs and CIP powder was studied using the Scanning Electron Microscope (SEM) (Zeiss EVO LS10; Cambridge, United Kingdom). Samples were fixed on stubs using double-sided adhesive carbon tape (SPI Supplies, West Chester, USA) and coated under vacuum with gold in a Q150R sputter coater unit from Quorum Technologies Ltd. (East Sussex, United Kingdom) in an argon atmosphere at 20 mA for 120 seconds.

#### 2.4.5. Transmission Electron Microscopy (TEM)

TEM (Philips, Tecrai10, Dutch) was used to examine the shape and surface morphology of the prepared CIP SLNs. CIP SLNs were dropped on copper grids natively stained by phosphotungstic acid and dried at room temperature.

#### 2.4.6. Differential Scanning Calorimetry (DSC) Studies

The thermal measurements of CIP powder and prepared SLN formulations were carried out by differential scanning calorimetry (Shimadzu DSC-60, Shimadzu Corporation, Tokyo, Japan). Samples (4–7 mg) were weighed and a heating rate of 10°C per minute in the range of 25°C–200°C was employed.

### 2.5. Release Studies

The study of CIP release kinetics from the prepared SLNs was performed using the dialysis bag technique [21]. Prior to the test, the dialysis bag (its molecular weight cut off: 12–14 kDa, Livingstone, NSW, Australia) was soaked in Milli Q double-distilled water for 12 h. Two mL aliquot of the prepared SLNs was placed inside the dialysis bag, tied at both ends, and dipped in 50 mL of release medium (phosphate buffered saline (PBS; 100 mM, pH 7.4)) in yellowish-brown colored glass bottles. The bottles were then placed in a thermostatic shaker at 37°C and operated at 100 rpm. At scheduled time intervals, 2 mL of the in vitro release medium was removed and replaced immediately with fresh medium. The amount of drug release was analyzed using HPLC.

### 2.6. Kinetic Studies

Zero-order, first-order, and Higuchi diffusion models and the Korsmeyer-Peppas equation were used to analyze the in vitro release data and to determine the best model that describes CIP release from SLNs. The best correlation coefficient value indicates the best release mechanism.

### 2.7. Statistical Analysis

The data obtained from the study was analyzed using analysis of variance (ANOVA) and *t*-tests to determine statistical significance by Graphpad Instat Version 3.05. A *P* value of <0.05 was considered statistically significant.

### 2.8. Stability Studies

The stability studies of the prepared ciprofloxacin solid lipid nanoparticles containing stearic acid (CIPSTE) were carried out by storage of 3 samples of SLNs in sealed yellowish-brown colored glass vials at room temperature (25°C) for 120 days. During this period, the particle size and particle size distribution were evaluated. At the end of the storage period the percent entrapment was calculated.

### 2.9. Antimicrobial Studies

The antimicrobial activity of CIP from the prepared CIPSTE formula was studied. Both the gram-negative pathogenic strain, for example,* P. aeruginosa*, and the gram-positive strain, for example,* S. aureus*, were used in this test. The bacterial suspension of each organism was speckled (100 *μ*L, concentration about 1 × 106 cfu/mL) individually by placing sterile swabs into a sterile Petri dish containing nutrient agar. Wells with 8 mm diameters on nutrient agar plates were done using a sterile cork borer. The control solution, blank SLNs, a physical mixture of CIP and blank SLNs, and CIPSTE SLNs (100 *μ*L) were poured into the wells. After a predetermined incubation time (6, 12, and 24 hours) at 37 ± 0.5°C, the inhibition zones around the walls were measured in mm using a caliper.

## 3. Results and Discussion

**Figure 1:**
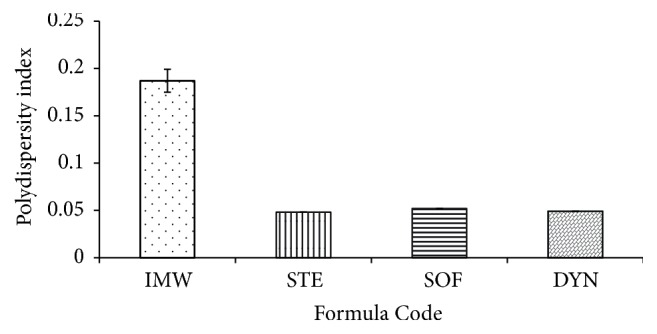
The mean polydispersity index for the investigated SLNs formulations.

**Figure 2:**
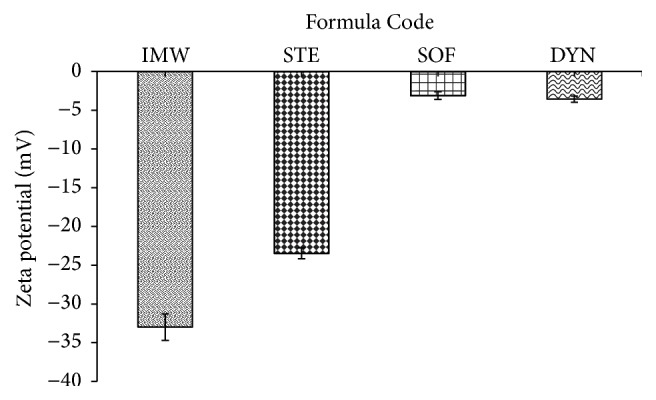
The mean zeta potential for the investigated SLNs formulations.

**Figure 3:**
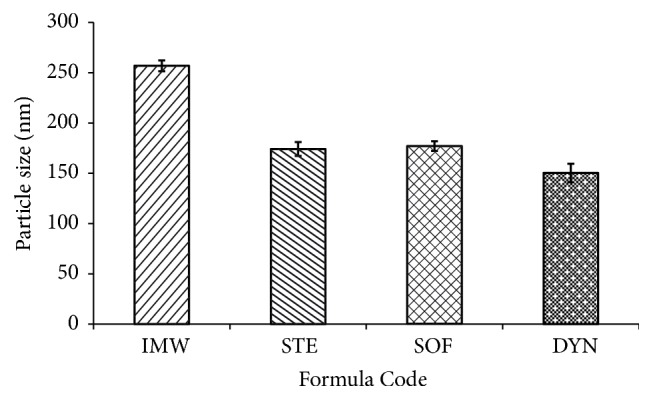
The mean particle size for the investigated SLNs formulations.

Figures 1, 2, and 3 exhibit the polydispersity, zeta potential, and the mean particle size of the prepared SLNs formulations, respectively. The polydispersity index can be used for measuring the uniformity of the particle size distribution of SLNs and the width of the particle dispersion. Polydispersity indices less than 0.3 are considered ideal and indicate a narrow size distribution [22, 23]. However, if the values of polydispersity are smaller than 0.1, the dispersion is considered as monodispersed [24]. It was found that all the prepared formulations had a polydispersity index of less than 0.1, indicating that the dispersions had a very narrow distribution and were monodispersed (Figure 1) (Table 2) [25]. Dynamic light scattering (DLS) was used to measure the particle size and size distribution of the prepared SLNs. The particle size of the prepared SLNs was found to range from 150.3 nm to 256.9 nm, and this depends on the type of the lipid used (Figure 3) (Table 2). It was found that all prepared SLNs samples showed a unimodal size distribution. The smallest mean particle size was obtained from DYN formula, which contains DYN (150 ± 9.2 nm), whereas the IMW formula showed the largest mean particle size (256.9 ± 5.4 nm). The same results are similar with that reported by Narala and Veerabrahma, 2013 [26].

There was statistically significant difference in the zeta potential of the prepared formulations (*P* < 0.05) using one-way analysis of variance (−32.99 mV for IMW formula, −23.47 mV for STE formula, −3.11 mV for SOF formula, and −3.57 mV for DYN formula) (Table 2) (Figure 2). By using the paired *t*-test, CIPSOF and CIPDYN formulae exhibited no statistically significant difference (*P* > 0.05).

**Figure 4:**
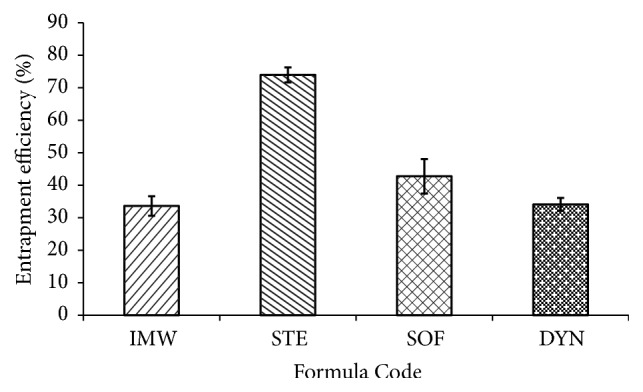
The % entrapment efficiency of the investigated SLNs formulations.

Drug loading, nanoparticle yield, and entrapment efficiency of CIP SLNs are summarized in Table 2. The lipid type used for preparing CIP SLNs had a significant effect on drug loading and the encapsulation efficiency of CIP SLNs. It was found that the CIPSTE formula showed the highest nanoparticle yield (73.66%), percent entrapment efficiency (73.94%) (Figure 4), and CIP loading (7.62%). On the other hand, the other formulae had low percentage of percent entrapment efficiency and percentage of CIP loading (Figure 4). This could be due to higher intactness of the solid lipid nanoparticles prepared from stearic acid [27].

**Figure 5:**
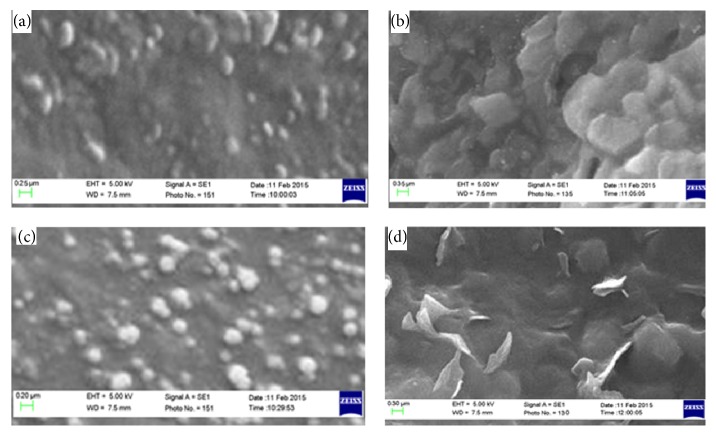
Scanning electron microscopic images of the investigated SLNs: (a) CIPIMW, (b) CIPSTE, (c) CIPDYN, and (d) CIPSOF.

Representative SEM photographic samples from the prepared CIP SLNs are exhibited in Figure 5. From these photographs, it was found that SLNs had a distinctive spherical shape with different sizes ranging from 150 up to 260 nm. This is consistent with the measurements of dynamic light scattering.

**Figure 6:**
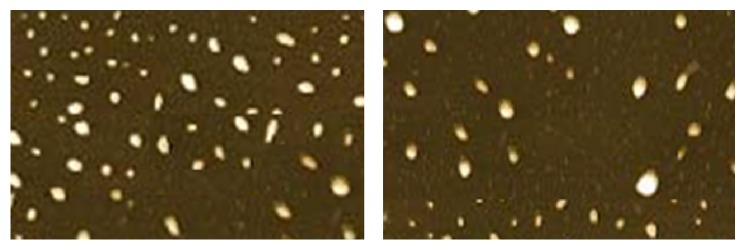
Atomic force microscopy images of CIPSTE solid lipid nanoparticles.

In order to study in detail the structure of SLNs, as the ability of SEM is limited for this reason, TEM was done. As shown in Figure 6, TEM micrographs of the prepared SLNs revealed that the prepared SLNs have a spherical shape with dimensions similar to those found through SEM and DLS measurements. When SLNs were examined by TEM, the outer shell of each nanoparticle appears as a dark region, and this indicates the occurrence of a crystalline region. On the other hand the bright region indicates amorphous phase.

**Figure 7:**
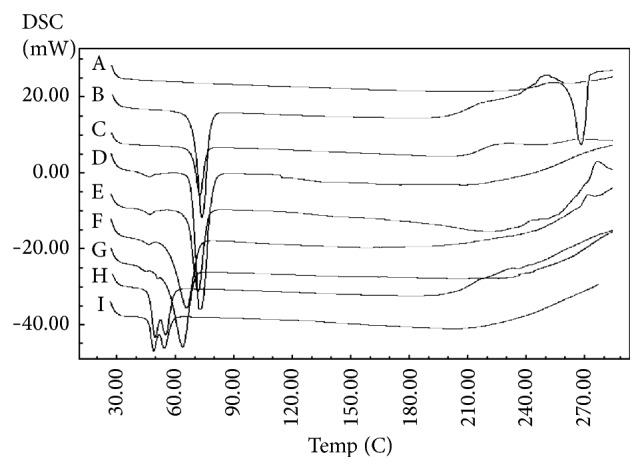
DSC thermogram of CIP, lipids, and SLNs: (A) CIP, (B) DYN, (C) CIPDYN, (D) STE, (E) CIPSTE, (F) IMW, (G) CIPIMW, (H) SOF, and (I) CIPSOF.

Crystallization, melting, and changing or modification in heat capacity of a substance can be suggested using DSC, which is also beneficial to evaluate not only the physicochemical properties of the encapsulated drug, but also the interaction among different compounds. The thermal behavior of the pure drug, IMW, SOF, DYN, and the different prepared SLNs formulations are presented in Figure 7. A sharp melting endotherm peak was seen with pure CIP at approximately 267.98°C with a heat of fusion of −276.05 (J/g). In addition, sharp endothermic peaks were exhibited at 73.57, 73.23, 65.94, and 49.83°C for DYN, STE, IMW, and SOF pure lipids with the heat of infusion of −267.7, −417.58, −270.76, and −172.33, respectively. From Figure 5, it was concluded that the specific peaks for the pure lipids appeared and the specific peak for CIP disappeared in all of the thermograms of the SLNs formulations. This confirms the solid crystalline state of these lipids inside the prepared formulations and explains complete solubilization of CIP in the lipid matrix of the prepared SLNs [28]. Moreover, the disappearance of the melting peak of CIP might be due to a reduction in the crystallinity of CIP or transformation to an amorphous state. These findings are similar to those reported by El-Badry and Fathy [29].

**Figure 8:**
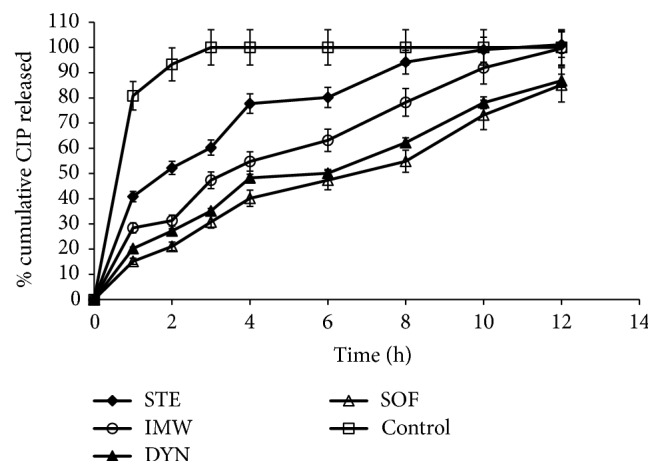
In vitro release of CIP from different SLNs in phosphate buffer pH 6.8 at 37°C.

Figure 8 contains the in vitro CIP release profile from the prepared SLNs in different lipids, that is, IMW, STE, SOF, and DYN, compared to the pure CIP solution in a phosphate buffer at a pH of 6.8 at 37°C. A burst drug release from the SLNs was exhibited after 1 hour. This burst release could be due to the rapid dissolution of drug molecules that are on the surface of the SLNs and existing independent of the surface layer of the particles. Then, the release rate became sustained for 12 hours. This sustained release of the drug might be attributed to the fact that the dispersed drug can only be released slowly from the lipid matrices through dissolution and diffusion [30]. On the other hand, the control (drug solution) could maintain this level of drug for only 3 hours. Formula CIPSTE that contains STE displayed the strongest burst effect and the most rapid release rate. Furthermore, formula CIPDYN, which contains DYN, showed a slow release profile with a low burst within the first hour. This could be due to the fast dissolution of CIP molecules existing in the surface layer of the SLNs, and this was more evident in the CIPSTE formula than the CIPDYN formula. This indicated that lipids with long chain triglycerides (DYN present in formula CIPDYN) transform slower than lipids with short chain triglycerides (IMW in formula CIPIMW, STE in formula CIPSTE, and SOF in formula CIPSOF), from the less stable form (*α*-form) to the more stable form (*β*-form) causing drug expulsion as it becomes more stable [31]. Previous studies indicated a biphasic drug release pattern with a high burst effect followed by a slow release profile in the SLNs [32, 33].

Table 3 represents the release data kinetics for the prepared SLNs. The data revealed a better fit to the Higuchi diffusion model. Analyzing the data for consecutive confirmation of the relative validity of the diffusion model was achieved using the equation of Korsmeyer et al. In addition, the release exponent (*n*) was calculated from the following equation where *Mt*/*M*_*∞*_ is the fraction released by the drug at time *t*, *K* is a constant incorporating structural and geometric characteristic, and *n* is the release exponent characteristic of the drug transport mechanism [34].

(4) $$\begin{array}{c} \frac{Mt}{M_{\infty }}=Kt^{n}, \end{array}$$

where *Mt*/*M*_*∞*_ is the fraction released by the drug at time *t*, *K* is a constant incorporating structural and geometric characteristic, and *n* is the release exponent characteristic of the drug transport mechanism.

If *n* = 0.43, Fickian diffusion is detected and the release rate depends on time (*t*). If 0.43 < *n* < 1.0, anomalous (non-Fickian) transport is indicated. If *n* = 1, the release is zero order. The results shown in the table indicate that the calculated n value was determined to be less than 0.43 in the case of the CIPSTE formula showing Fickian diffusion, and the release was dependent on time (*t*). However, *n* values for other formulae (CIPIMW, CIPSOF, and CIPDYN) were more than 0.43 and less than one, and this indicates anomalous (non Fickian) transport. This could be due to the fact that the diffusion refers to combination of both diffusion and erosion controlled rate release [35, 36].

**Figure 9:**
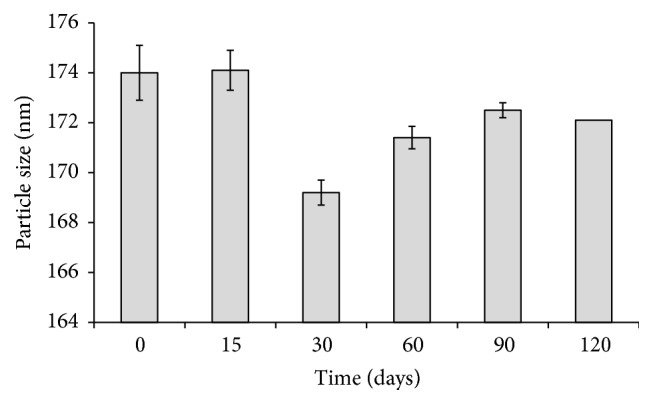
Particle size of CIPSTE formula stored at room temperature over a period of 120 days.

**Figure 10:**
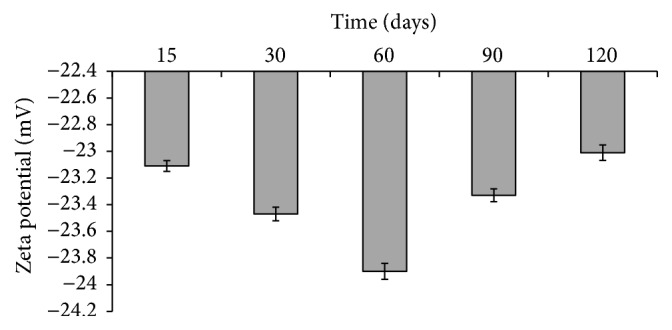
Zeta potential of CIPSTE formula stored at room temperature over a period of 120 days.

Figures 9 and 10 show the particle size and zeta potential of the investigated CIPSTE SLNs stored at room temperature for a period of 120 days. It found no significant difference in particle size and zeta potential, as the percent change is around 100%. Concerning the particle size, it was noted that the particle sizes ranged between 169 and 174 nm, and this indicates the stability of the particle size and hence the SLNs (Figure 9) (Table 4). Regarding the zeta potential of the CIPSTE formula, it was intriguing that the zeta potential was almost unchanged (Figure 10) (Table 4). Throughout the period of storage, the CIPSTE formulation exhibited no phase separation, no creaming, no color change, and no gelling. At the end of study period, the entrapment efficiency was determined and was 70.33%. This slight lowering in entrapment efficiency might be due to slow transitions of lipid from metastable forms to stable forms on storage. Therefore, SLNs are considered suitable carriers [37].

**Figure 11:**
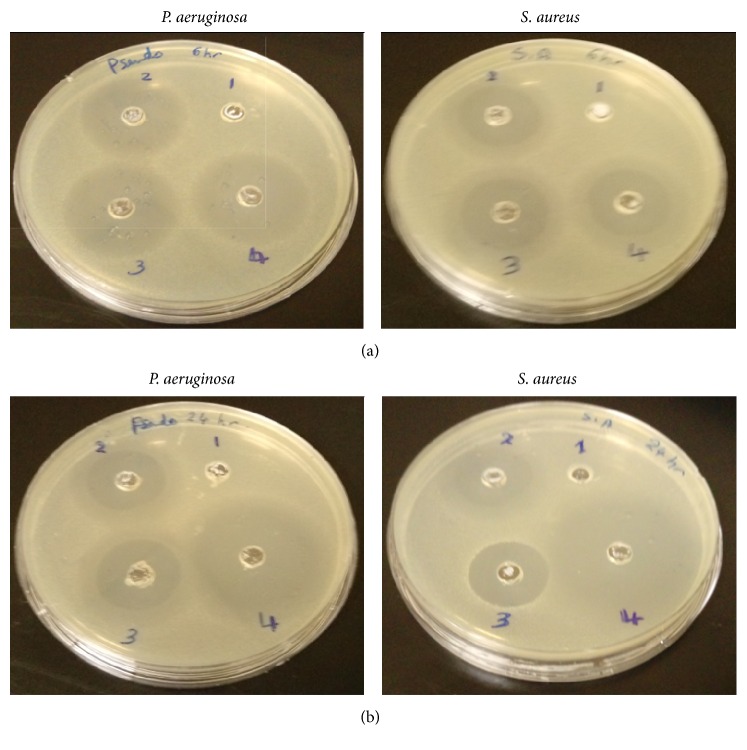
Zone of inhibition of CIPSTE formula (4) compared to SLNs (1), control (2), and physical mixture samples (3) after 6 (a) and 24 (b) hours for* P. aeruginosa* and* S. aureus* bacterial strains.

The zone of inhibition was determined and plotted against bacterial strains as shown in Figure 11. The results confirmed that there is no inhibition zone with the blank SLNs which were free of CIP. The inhibitory zone of CIP in the CIPSTE formula was significantly higher than other samples in both* P. aeruginosa* and* S. aureus* cases. In comparing the inhibitory zone of CIP in the CIPSTE formula for the two bacterial strains, it was concluded that CIP exhibits an inhibitory zone with* P. aeruginosa* and* S. aureus*. This could be due to the fact that CIP is more strongly effective on gram-negative than gram-positive bacteria [38, 39]. The results showed that the control solution (free CIP) and physical mixture of CIP exhibited a maximum inhibition zone after 6 hours in the case of* P. aeruginosa* and* S. aureus*. However, the CIPSTE formula increasingly inhibited the growth of bacteria for both* P. aeruginosa* and* S. aureus*. These results indicated that CIP could inhibit the growth of bacteria for a longer time period than free CIP and blank. This indicated that CIP-loaded SLNs have superior antibacterial activity related to free CIP. The reasons for these results could be due to the lipophilic nature of STE that improve the cellular entering of CIP into the bacterial membrane and the small size of the particles.

## 4. Conclusions

The purpose of this study was to prolong the therapeutic effect of CIP by loading CIP into SLNs using different lipids and the ultrasonic melt-emulsification method. It was concluded that the optimum formulation was CIPSTE, which exhibits a relatively low polydispersity index, low particle size, high zeta potential, and high percentage entrapment efficiency. Moreover, the CIPSTE formula showed not only the optimum drug release pattern, but also superior antibacterial activity. Accordingly, it could be decided that CIP might be administered at longer time intervals by loading it in a solid lipid nanoparticle formulation.

## Figures and Tables

**Table 1:** Composition of the prepared solid lipid nanoparticle formulations.

Formulation code	CIP (mg)	Lipid			Tween 80 (mg)	Sodium deoxycholate (mg)
		Type	Melting point (°C)^*∗*^	(mg)		
CIPIMW	50	IMW	~61	450	45	22.5
CIPSTE	50	STE	70	450	45	22.5
CIPSOF	50	SOF	55–60	450	45	22.5
CIPDYN	50	DYN	70–74	450	45	—

CIPIMW Ciprofloxacin solid lipid nanoparticles containing Imwitor.

CIPSTE Ciprofloxacin solid lipid nanoparticles containing Stearic acid.

CIPSOFT Ciprofloxacin solid lipid nanoparticles containing Softisan.

CIPDYN Ciprofloxacin solid lipid nanoparticles containing Dynasan.

^*∗*^Vladimir Torchilin. *Handbook of Nanomedical Research*: *Fundamentals, Applications and Recent Developments*, *Materials for Nanomedicine,Vol. 3*.

**Table 2:** Percent drug loading measurements for all formulations.

Parameter	Formula Code			
	CIPIMW	CIPSTE	CIPSOF	CIPDYN
Drug loading (%)	3.51 ± 0.31	7.62 ± 0.07	4.08 ± 0.09	3.11 ± 0.03
Nanoparticle yield (%)	64.33 ± 1.51	73.66 ± 1.81	68.95 ± 2.01	71.24 ± 1.09

**Table 3:** Release kinetic data of the investigated CIP SLNs.

Formula Code	Zero-order model	First-order model	Higuchi diffusion model	Peppas model	*N* ^*∗*^
	*r*	*r*	*r*	*r*	
CIPSTE	0.908307305	0.976226353	0.989261212	0.986430942	0.40488
CIPIMW	0.954829809	0.988204124	0.992688565	0.97344802	0.510222
CIPSOF	0.968285237	0.989224718	0.990192679	0.991251705	0.648841
CIPDYN	0.950442524	0.978375342	0.990584143	0.985940377	0.549895

**Table 4:** Particle size and zeta potential of the investigated CIPSTE formula stored at room temperature at several time points.

Time (days)	Particle size		Zeta potential (mV) ± SD^*∗*^	
	Value (nm) ± SD^*∗*^	% change	Value (mV) ± SD^*∗*^	% change
0	174 ± 0.9		−23.4 ± 0.02	
15	174.1 ± 1.1	1.0006	−23.1 ± 0.04	0.9876
30	169.2 ± 0.8	0.9724	−23.47 ± 0.05	1.01558
60	171.4 ± 0.5	0.9851	−23.90 ± 0.06	1.0183
90	172.5 ± 0.45	0.9914	23.33 ± 0.048	0.9762
120	172.2 ± 0.3	0.9891	−23.01 ± 0.058	0.9863

^*∗*^Measurement of 3 samples.
