# Corrigendum to “Ciprofloxacin Controlled-Solid Lipid Nanoparticles: Characterization, In Vitro Release, and Antibacterial Activity Assessment”

**DOI:** 10.1155/2017/6761452

**Published:** 2017-08-13

**Authors:** Gamal A. Shazly

**Affiliations:** ^1^Department of Pharmaceutics, College of Pharmacy, King Saud University, P.O. Box 2457, Riyadh 11451, Saudi Arabia; ^2^Department of Industrial Pharmacy, Faculty of Pharmacy, Assiut University, Assiut 71526, Egypt

In the article titled “Ciprofloxacin Controlled-Solid Lipid Nanoparticles: Characterization, In Vitro Release, and Antibacterial Activity Assessment” [1], there was an error in Acknowledgments, which should be corrected as follows.

The author acknowledges the Deanship of Scientific Research at King Saud University for funding this work through the Research Group Project no. RGP-VPP-139. The author also extends gratitude to Dr. Amany Z. Mahmoud, Department of Pharmaceutics, College of Pharmacy, King Saud University, for her technical support in the antibacterial activity study.
